# Co-occurrence of lung adenocarcinoma with rapidly progressive dementia and multiple cerebral microbleeds: a case report

**DOI:** 10.1186/s13256-026-05846-x

**Published:** 2026-02-02

**Authors:** Jinyi Yan, Peicai Fu, Chengqing Yang, Ya Xu, Qiliang Liu, Kalam Choi, Zhijun Li

**Affiliations:** 1 Department of Neurology, Tongji Hospital, Tongji Medical College, Huazhong University of Science and Technology, Wuhan, 430030 Hubei China; 2 Department of Pulmonary and Critial Care Medicine, Wuhan Pulmonary Hospital (Wuhan Institute for Tuberculosis Control), Wuhan, 430030 Hubei China; 3 Department of Pathology, Wuhan Pulmonary Hospital (Wuhan Institute for Tuberculosis Control), Wuhan, 430030 Hubei China

**Keywords:** Rapid progressive dementia, Lung adenocarcinoma, Cerebral microbleeds, Cerebral amyloid angiopathy, Acute hemorrhagic leukoencephalitis

## Abstract

**Background:**

Various neurological diseases can cause rapidly progressive dementia, which is a clinical syndrome characterized by a rapid decline in cognitive function over a short period, typically less than 1 or 2 years. It can be caused by various neurological diseases, including neurodegenerative, inflammatory, vascular, metabolic, and neoplastic central nervous system diseases. Rapidly progressive dementia is particularly associated with Creutzfeldt–Jakob disease, but other conditions such as immune-mediated encephalitis, rapidly progressive subtypes of Alzheimer’s disease, and various other mimics of prion diseases must also be considered. Multiple cerebral microbleeds are typical imaging features of cerebral small vessel diseases, but can also appear in other rare conditions.

**Case presentation:**

We describe the case of a 45-year-old Asian female patient with lung adenocarcinoma who exhibited rapidly progressive dementia and multiple cerebral microbleeds. The patient was a 45-year-old woman who experienced rapid cognitive decline without obvious triggers, accompanied by disorganized speech, difficulty in expression, and short-term memory loss. Brain magnetic resonance imaging revealed widely distributed microhemorrhages, while computed tomography and pathological examination further confirmed the diagnosis of lung adenocarcinoma. The patient did not undergo a brain biopsy because of the rapid deterioration of her illness. Her condition deteriorated rapidly, leading to death in the fourth month.

**Conclusion:**

Herein, we discuss the presence of the apolipoprotein ε4 allele risk gene and the role of the tumor in causing multiple nodular lesions in the patient’s brain, as well as multiple microbleeds. The role of the apolipoprotein ε4 allele risk gene in multiple nodular lesions of the brain and CMB requires further study, as it may be responsible for the rapid cognitive decline and imaging findings observed in patients. The role of the tumor in causing these brain lesions and cerebral microbleeds is also of interest as it may help to provide insight into the pathophysiological mechanisms of rapidly progressive dementia in the context of cancer. This case highlights the need for a comprehensive diagnosis, including magnetic resonance imaging, blood and cerebrospinal fluid analyses, and brain biopsy, to identify treatable causes of rapidly progressive dementia.

**Supplementary Information:**

The online version contains supplementary material available at 10.1186/s13256-026-05846-x.

## Introduction

Rapidly progressive dementia (RPD) develops quickly, with the interval from the first symptom to the onset of dementia ranging from weeks to months. The causes of RPD include a range of neurological diseases that require systematic examination and evaluation. Multiple cerebral microbleeds (CMBs) are typical imaging manifestations associated with cerebral small vessel disease, commonly identified in the elderly, as well as patients with dementia, hypertension, arterial disease, or cerebral amyloid angiopathy (CAA), and so on. [1]. However, this phenomenon may also be linked to other less common processes or conditions, such as coronavirus disease 2019 (COVID-19)-related demyelinating hemorrhagic leukoencephalitis [2, 3], rare tumor complications [4], and tumor hemorrhagic metastasis, which involves various triggering factors. Herein, we present a case of lung adenocarcinoma manifesting as RPD complicated by multiple CMBs to raise awareness of this condition.

## Case

A 45-year-old Asian female patient was admitted to our hospital on 11 June 2024. After catching a cold in mid-April, she began sleeping for 18–20 hours daily, but was able to engage in normal activities when awake. A month later, she presented with slowed reactions and was involved in a minor car accident due to delayed responses, although she did not sustain any injury. Her symptoms worsened thereafter, including confused speech, difficulty expressing herself, and memory issues, such as decreased short-term memory, reduced calculation ability, and impaired orientation to time and place, along with a depressive mood, without hallucinations or delusions. Throughout her illness, she experienced no fever, dizziness, headache, bowel or bladder dysfunction, or limb movement disorders. Her medical history included 5 years of well-controlled hypertension, and several years of untreated chronic hepatitis B infection. She was married and resides with family members, enjoyed good social support, and had no known occupational exposure history related to lung cancer. She had no family history of dementia or other significant personal history, and her menstrual cycle was normal.

Upon admission, the patient’s consciousness was clear, but the content of her speech was confusing, with sluggish responses during communication, decreased memory, abnormal orientation to time and place, reduced calculation ability, slowed speech, and no significant speech articulation disorders. Neurocognitive testing revealed a Mini-Mental State Examination (MMSE) score of 13/30, Montreal Cognitive Assessment (MOCA) score of 4/30, and a modified Rankin scale (mRS) score of 3. The muscle strength and tone in the limbs were normal, with positive pathological signs on the left side. Neck stiffness was also observed. The patient exhibited comprehensive cortical dysfunction, mainly manifesting as RPD, accompanied by impairments in emotions, language, and sleep cycles. However, at this stage, the etiology remained undetermined.

Routine serological investigations, including metabolic indicators and systemic autoimmune diseases (such as the vasculitis-related antibody spectrum), were normal. Tests revealed mildly elevated high-sensitivity C-reactive protein (CRP 1.5 mg/L↑, range < 1 mg/L) and D-dimer levels (1.44 µg/mL↑, range < 0.5 mg/L). Hepatitis B DNA was significantly elevated, indicating active hepatitis (1000.00 IU/mL↑, range < 500.00 IU/mL). Blood ammonia was within the normal range, while an abdominal computed tomography (CT) scan was generally normal, with no significant abnormal signals observed. Other infection indicators [erythrocyte sedimentation rate (ESR) and procalcitonin] and pathogen tests [tuberculosis infection T-cell test, respiratory pathogens, hepatitis C virus, syphilis, human immunodeficiency virus (HIV)] revealed no abnormalities. However, assessment of dementia-related risk genes, including apolipoprotein (APOE) gene typing, revealed the ε4/ε4 type.

**Fig. 1:**
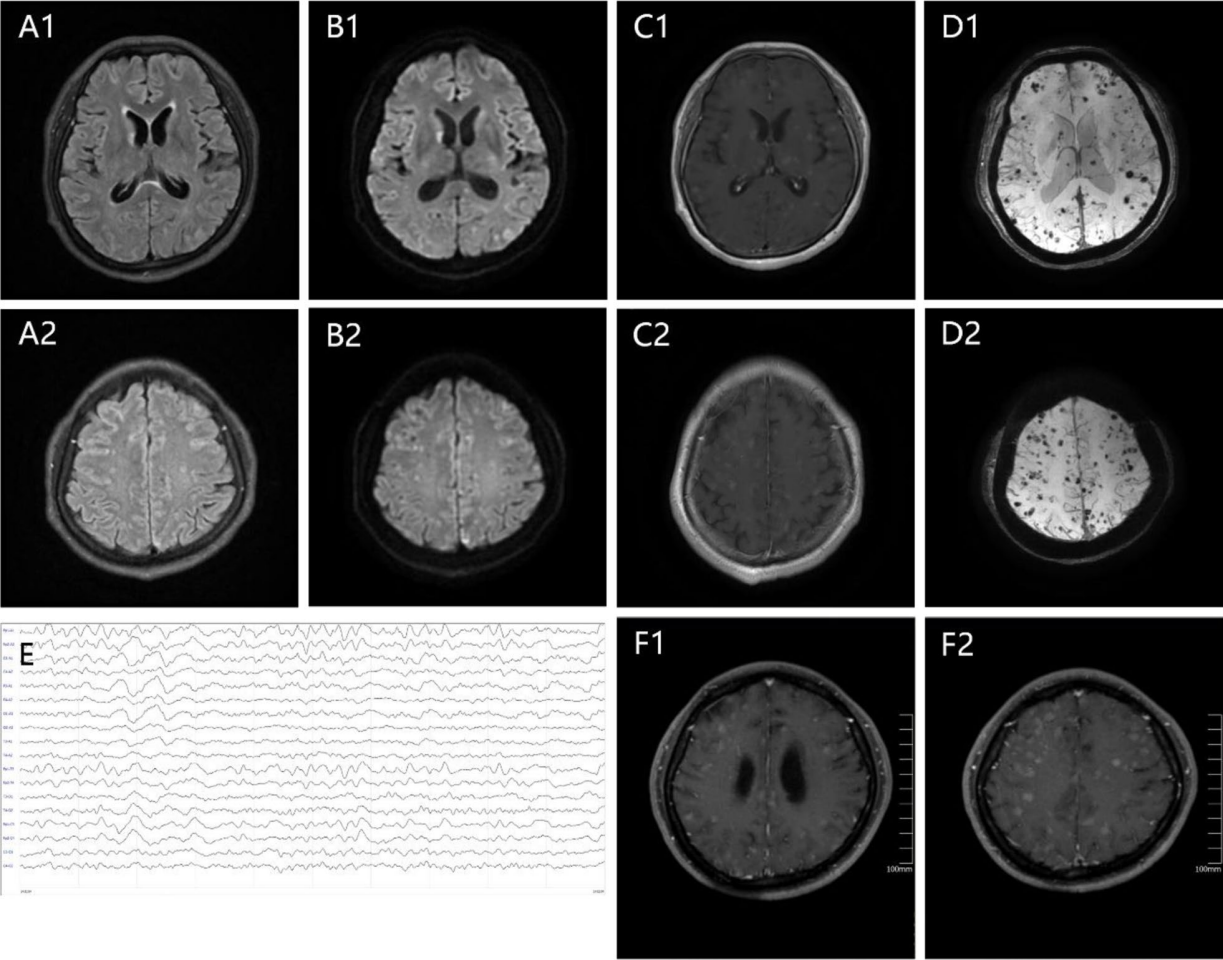
**A**–**D** multiple round abnormal signals distributed in the bilateral frontal, parietal, temporal, and occipital lobes; semi-oval center; basal ganglia region; brainstem; and cerebellum, with diameters of approximately 3–5 mm. A1–A2: T2 flair showing longer T2 signals; B1–B2: Diffusion-weighted imaging (DWI) showing some lesions with restricted diffusion; C1–C2: lesions showing nodular or ring-like enhancement; D1–D2: Susceptibility-weighted imaging (SWI) showing multiple cerebral microbleeds; **E**: 24-hour video electroencephalogram showing widespread slow waves; **F**: T1 enhances showing lesion progression compared with the previous images

Brain magnetic resonance imaging (MRI) revealed multiple round abnormal signals distributed in the bilateral frontal, parietal, temporal, and occipital lobes; semi-oval center; basal ganglia region; brainstem; and cerebellum, with diameters of approximately 3–5 mm. These lesions exhibited slightly shorter T1 and slightly longer T2 signals, with some showing restricted diffusion and nodular or ring-like enhancement, in addition to significant internal bleeding (Fig. 1A–D). No abnormalities were noted in the large cerebral blood vessels on computed tomography angiography (CTA). Cardiac enzyme levels, electrocardiography, cardiac ultrasound, and lower-limb vascular ultrasound all revealed no abnormalities. However, 24-hour video electroencephalogram showed widespread slow waves, with no observed epileptiform discharges (Fig. 1E).

Analysis of cerebrospinal fluid (CSF) was conducted to further detect intracranial infections and immune-related lesions. The lumbar puncture pressure and white blood cell count in the CSF were normal; however, an elevated total protein level (815 mg/L) was observed. The CSF immunoglobulin (Ig)G was 92.6 mg/L↑, and the IgG synthesis rate in the CSF over 24 hours was 19.67 mg/24 hours↑. Oligoclonal bands were negative. All CSF tests were negative (including bacteria, fungi, tuberculosis, and other pathogens). Cytopathological examination of the CSF revealed no abnormal morphological cells, while blood and CSF cultures were sterile.

**Fig. 2:**
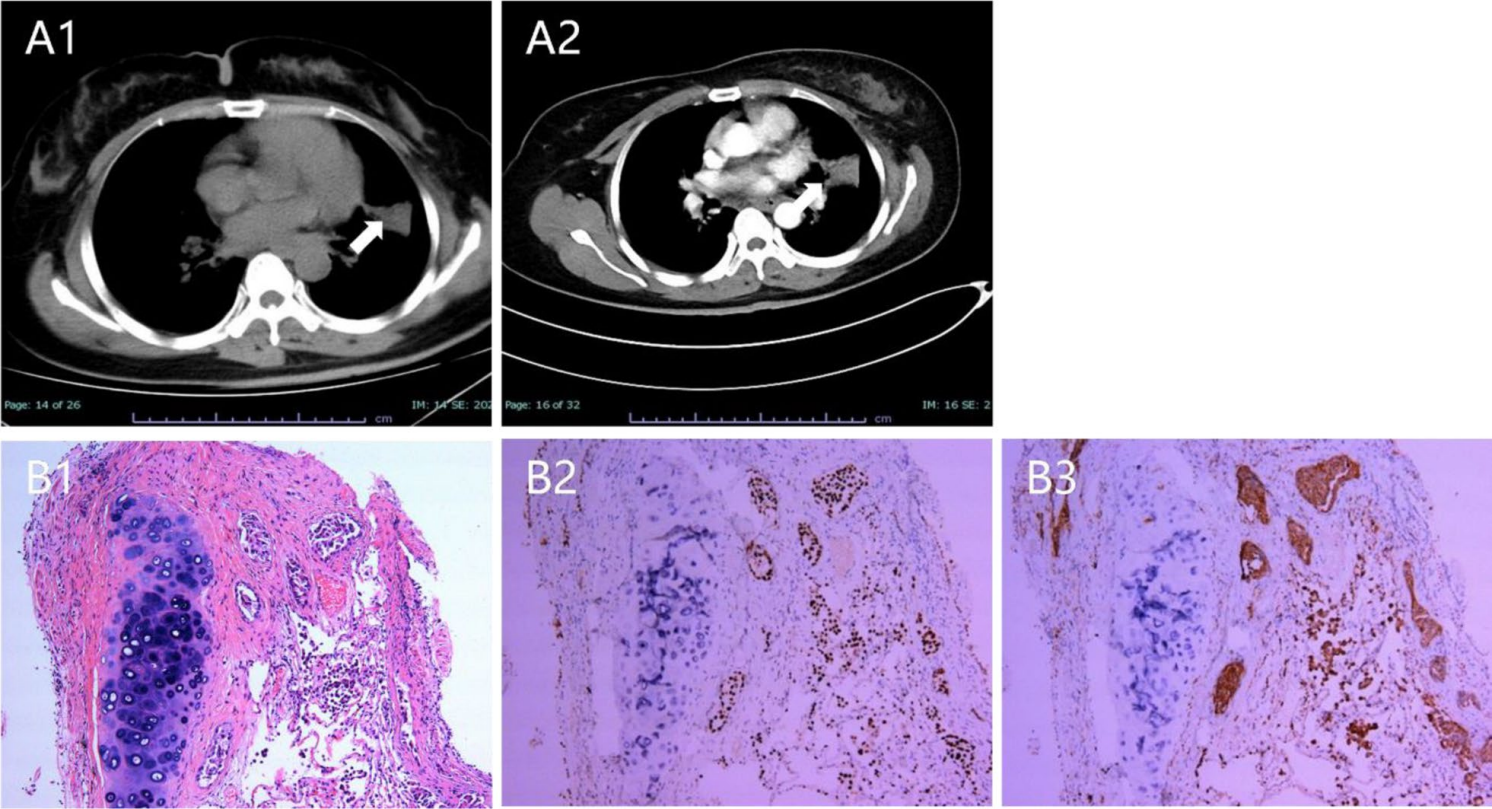
A1–A2: pulmonary computed tomography scan showing a mass-like lesion in the posterior segment of the left upper lobe, measuring approximately 49.2 mm × 30.5 mm, with uneven enhancement on the enhanced scan. B1 hematoxylin and eosin staining revealed irregular glandular infiltrative growth in the mucosal stroma with eosinophilic glandular epithelial cytoplasm and the nuclei, showing deep staining with atypia. Additionally, scattered growth of micropapillary epithelial cell clusters was observed near the alveolar lumen; B2–B3: immunohistochemical staining was positive for Napsin A and TTF-1

Ultrasound of the uterine adnexa performed to screen for tumors revealed no abnormalities. The carcinoembryonic antigen (CEA) level was elevated, reaching 22.08 ng/ml↑ (normal range < 5.0 ng/ml). A pulmonary CT scan further revealed a mass-like lesion in the posterior segment of the left upper lobe, measuring approximately 49.2 mm × 30.5 mm, with uneven enhancement on the enhanced scan. Interstitial changes, bilateral pleural thickening, and adhesions were also observed, along with enlargement of the supraclavicular lymph nodes (Fig. 2A1-2). Further details are provided in the Supplementary Figures.

Further, pathological biopsy of the left supraclavicular lymph nodes indicated reactive hyperplastic lymphadenitis. The patient refused further pulmonary biopsy and was discharged with prednisone (60 mg/day). However, the patient’s symptoms worsened after discharge. Approximately 80 days following disease onset, the patient exhibited altered consciousness, drowsiness, and response to calling, but was unable to communicate. The patient was unable to cooperate during the limb strength examination. Increased muscle tone was noted in the limbs with abnormal flexion contractures, while significantly elevated muscle tone was observed in both upper limbs. Babinski’s sign was positive. Neck stiffness was also observed. Reexamination of the ESR and procalcitonin (PCT) levels revealed no abnormalities. Routine CSF examination also revealed no abnormalities, while the cell smear was unremarkable. Rescreening for pathogens in both the serum and CSF yielded negative results, and the second CSF culture was negative.

Brain MRI revealed lesion progression compared with previous scans (Fig. 1F1–2). CSF CEA level was significantly elevated at 81.59 ng/mL. Furthermore, ultrasound bronchoscopy with biopsy and a lung tissue biopsy confirmed the diagnosis. Hematoxylin and eosin (H&E) staining (Fig. B1) revealed irregular glandular infiltrative growth in the mucosal stroma, with eosinophilic glandular epithelial cytoplasm, while the nuclei showed deep staining with atypia. In addition, scattered growth of micropapillary epithelial cell clusters was observed near the alveolar lumen. Immunohistochemical staining revealed the following: CK7 (+), Ki67 (approximately 1%), Napsin A (+), P40 (−), TTF-1 (+) (Fig. 2B1–3). The tumor was positive for the EGFR L858R mutation (class 1). The final diagnosis of the pulmonary lesions was non-small cell lung cancer (lung adenocarcinoma). A concise timeline summarizing the patient’s key clinical events, investigations, and interventions is provided in Table 1.

**Table 1:** Timeline of patient’s rapid clinical course

Time	Clinical events, findings, and interventions
Mid-April 2024 (T = 0)	Catches a cold → begins sleeping 18–20 hours per day but maintains normal daytime activity
Mid-May 2024 (~1 month after onset)	Slowed reactions; minor car accident due to delayed responses; no injury. Cognitive decline progresses: confused speech, difficulty expressing herself, impaired memory, calculation, orientation; depressive mood; no fever/headache or limb deficits
11 June 2024 (hospital admission)	Clear consciousness but confused speech; slow responses; comprehensive cortical dysfunction with rapidly progressive dementia and impairments in emotion, language, and sleep cycles; MMSE 13/30, MoCA 4/30; neck stiffness; left pathological signs
11–15 June 2024	Routine labs mostly normal; mild ↑ CRP and D-dimer; active HBV (HBV-DNA ↑); APOE ε4/ε4 detected; abdominal CT normal. MRI: multiple 3–5 mm lesions (frontal, parietal, temporal, occipital, basal ganglia, brainstem, cerebellum) with diffusion restriction, nodular/ring enhancement, intralesional hemorrhage. CTA: normal
Mid–late June 2024	24-hour EEG: diffuse slowing, no epileptiform discharges. CSF: normal pressure/WBC; ↑ protein (815 mg/L); ↑ IgG and IgG synthesis rate; OCB negative; all microbiological tests negative.Tumor screening: CEA ↑ (22.08 ng/mL). Chest CT: left upper-lobe mass (49 × 31 mm), heterogeneous enhancement; interstitial changes; supraclavicular lymphadenopathy. Node biopsy: reactive hyperplasia
Late June 2024 (hospital discharge)	Patient refuses lung biopsy. Discharged with prednisone 60 mg/day
July 2024 (~80 days from onset)	Symptoms worsen: drowsiness, impaired communication; unable to cooperate with motor exam; increased limb tone with flexion contractures. Lab: ESR/PCT normal; repeat CSF normal; pathogen panel negative
Late July 2024	MRI: progression of brain lesions. CSF CEA ↑ to 81.59 ng/mL
Early August 2024	Bronchoscopic biopsy + lung tissue biopsy: adenocarcinoma confirmed (CK7 +, Napsin A +, TTF-1 +, EGFR L858R mutation)
August 2024	Targeted therapy with amivantamab initiated; entecavir started for HBV
Late August–Early September 2024 (fourth month)	Rapid deterioration despite treatment → death in the fourth month of disease course

Subsequently, the patient received targeted therapy with amivantamab and antiviral treatment with entecavir for hepatitis B. However, her condition unfortunately worsened rapidly, with significant deterioration resulting in death in the fourth month of the disease course.

## Discussion

The main symptoms exhibited by this patient in the early stages of the disease included subacute onset and rapidly progressive cognitive dysfunction, accompanied by alterations in sleep cycles. Due to the wide range of potential causes, screening for the etiology of rapidly progressive dementia is commonly organized using the mnemonic device Vascular, Infectious, Toxic-metabolic, Autoimmune/inflammatory, Metastases/neoplastic, Iatrogenic, Neurodegenerative, or Systemic/seizures/structural (VITAMINS) [5].

Although the patient had a history of hypertension, her blood pressure was well controlled, and the distribution of the imaging lesions did not match the pattern of the large vessel lesions. Additionally, no abnormalities were detected in the cerebral vessels using CTA; therefore, large cerebral vessel diseases were not considered. No embolic vascular disease was observed. The patient’s D-dimer level ruled out the possibility of DIC. There were no positive findings of systemic connective tissue disease or metabolic factors. Multiple serological tests and CSF examinations revealed no evidence of infectious pathogens, and the patient did not exhibit fever or symptoms related to infection, thus allowing for the exclusion of infection-related causes. Meanwhile, the progression of the disease, along with the EEG and brain MRI, also did not support the diagnosis of sporadic Creutzfeldt–Jakob disease (CJD). Although the patient was in the active phase of hepatitis B, her blood ammonia level was normal, while liver function, other serological indicators, and liver imaging examinations did not support a diagnosis of hepatic encephalopathy.

Considering the patient’s rapidly progressive clinical symptoms, lung tumor condition, and brain imaging findings, paraneoplastic autoimmune disorders or tumor metastasis were considered as the key differential diagnoses. According to the latest diagnostic criteria for paraneoplastic neurological syndrome (PNS), limbic encephalitis is defined as a high-risk clinical phenotype of PNS [6], while such cases are often reported in relation to lung cancer. However, imaging revealed multiple nodular lesions and microhemorrhages with enhancement signals that did not match the common patterns of limbic encephalitis, such as medial temporal lobe T2-hyperintense signals [7].

Acute hemorrhagic leukoencephalitis (AHLE) was observed in a patient with lung adenocarcinoma, confirmed by postmortem examination [4]. AHLE is a rare and severe form of acute disseminated encephalomyelitis (ADEM) that has been recently reported in some cases of COVID-19 [8]. The common clinical manifestations of AHLE include acute encephalopathy and rapid neurological deterioration, resulting in coma and even death. Prominent pathological features include acute perivenous hemorrhagic encephalitis in multiple brain areas, perivascular demyelination with some loss of axis cylinders, and ball-and-ring hemorrhages, while the cerebral cortex and basal ganglia are spared [4]. Typical neuroimaging characteristics of AHLE include multifocal, variably sized, and poorly defined white matter lesions with cortical sparing. The brainstem, cerebellar peduncles, and deep grey matter may also be involved. The lesions appear hyperintense on T2-weighted (T2W) and fluid-attenuated inversion recovery (FLAIR) images, and hypointense on T1-weighted (T1W) images, and show microhemorrhages, varying degrees of restricted diffusion, and enhancement following contrast administration [8]. In the present case, the pattern of rapid disease progression suggested the possibility of AHLE. Unfortunately, we did not perform a brain biopsy to confirm the diagnosis. The diagnosis of AHLE presents several ongoing challenges, primarily due to imaging findings in patients with cortical involvement characterized by T2 hyperintensity and enhancement after contrast, along with localized microhemorrhages and elevated levels of CEA in the CSF. Meningeal metastases of lung adenocarcinoma should also be considered in conjunction with persistent signs of neck stiffness.

The APOE ε4 allele has been found to correlate with increased amyloid accumulation in the parenchymal and meningeal cerebrovasculature [9, 10], and significantly increases the carrier’s risk of developing Alzheimer’s disease (AD) [11]. CAA can cause small infarcts visible on diffusion-weighted imaging in approximately 30% of patients with transient focal neurological episodes [12]. What distinguished our case from classic CAA was the rapid progression of the disease, with progression to cognitive decline to consciousness impairment and death occurring within 4 months. Additionally, the patient did not have any prior episodic clinical history of significant vascular events, nor was there any evidence in the imaging findings. Furthermore, the modified Boston criteria indicated that CAA-related CMBs are only distributed in the cortical–subcortical regions of the brain, are commonly found in the posterior brain areas (occipital and parietal lobes), and do not involve the basal ganglia [13]. However, in our case, in addition to the cortical–subcortical regions of the brain, imaging revealed widespread involvement of the basal ganglia, brainstem, and cerebellum. Interestingly, a recent small-cell lung carcinoma patient presented with two coexisting brain disorders: paraneoplastic encephalomyelitis and CAA [14]. Biopsy is the only method used to obtain a definitive diagnosis of rare comorbidities.

CAA can trigger an autoimmune response; as such, CAA-related inflammation (CAA-ri) was considered as a possible diagnosis in our case. CAA-ri is characterized by the deposition of Aβ (amyloid-β) in the media and adventitia of cortical and leptomeningeal vessels, along with a perivascular, nondestructive accumulation of inflammatory cells [15]. Approximately 34% of patients with CAA-ri have the homozygous ε4 allele. The most common clinical features of CAA-ri are cognitive decline, asymmetric T2/FLAIR high-signal intensity lesions in the white matter, and MCBs in the brain [15]. However, CAA-ri is a diagnosis of exclusion. In our case, owing to the presence of a tumor, the ineffectiveness of prednisone therapy, and imaging changes, it was challenging to establish a diagnosis of CAA-ri.

Additionally, other etiologies of MCBs should be differentiated from radiologically similar diseases, such as small cavernous hemangioma and CADASIL. However, the presence of an associated familial history, chronic disease course, and other concurrent symptoms allow for a correct diagnosis.

In conclusion, because of the absence of a brain biopsy and the rapid progression of the patient’s clinical symptoms, auxiliary examinations, and intracranial imaging characteristics, we ultimately deduced that the most likely main reasons for the disease progression and the patient’s death were a paraneoplastic autoimmune process and multiple metastases from pulmonary adenocarcinoma (in the brain parenchyma and meninges). Further, the presence of ε4 allele risk genes and the role of tumors contribute to the occurrence of multiple nodular lesions as well as CMBs in the patient’s brain.

## Conclusion

This case describes a patient with rapidly progressive dementia accompanied by widespread cerebral microbleeds and multiple intracranial nodular lesions in the context of lung adenocarcinoma. The rapid neurological deterioration, elevated CSF CEA levels, and progression of MRI abnormalities indicated a malignant-related neurological process involving the brain parenchyma and meninges. The coexistence of the APOE ε4/ε4 genotype may have been associated with increased susceptibility to microvascular pathology. The findings suggest that both tumor-associated mechanisms and genetic risk factors may contribute to the imaging and clinical manifestations. This case emphasizes the diagnostic complexity of RPD and demonstrates the value of integrating neuroimaging, laboratory testing, and tumor evaluation when assessing rapidly progressive cognitive decline.

## Supplementary Information

Supplementary material 1.

## Data Availability

Not applicable.

## References

[1] Haller S, Haacke EM, Thurnher MM, *et al*. Susceptibility-weighted imaging: technical essentials and clinical neurologic applications. Radiology. 2021;299(1):3–26. 33620291 10.1148/radiol.2021203071

[2] Grzonka P, Scholz MC, De Marchis GM, *et al*. Acute hemorrhagic leukoencephalitis: a case and systematic review of the literature. Front Neurol. 2020. 10.3389/fneur.2020.00899. 32973663 10.3389/fneur.2020.00899 PMC7468463

[3] Manzano GS, McEntire CRS, Martinez-Lage M, et al. Acute disseminated encephalomyelitis and acute hemorrhagic leukoencephalitis following COVID-19 . Neurol Neuroimmunol Neuroinflammation, 2021, 8(6). 10.1212/NXI.0000000000001080 PMC8404207 34452974

[4] Mitsuyama Y, Fujimoto S, Kohno T. An autopsied case of acute hemorrhagic leukoencephalitis. Jpn J Psychiatry Neurol. 1986;40(1):105–12. 3773347 10.1111/j.1440-1819.1986.tb01617.x

[5] Day GS. Rapidly progressive dementia. Continuum (Minneap Minn). 2022;28(3):901–36. 35678409 10.1212/CON.0000000000001089 PMC9580391

[6] Graus F, Vogrig A, Muñiz-Castrillo S, *et al*. Updated diagnostic criteria for paraneoplastic neurologic syndromes. Neurol Neuroimmunol Neuroinflamm. 2021. 10.1212/NXI.0000000000001014. 34006622 10.1212/NXI.0000000000001014 PMC8237398

[7] Casagrande S, Zuliani L, Grisold W. Paraneoplastic encephalitis. Paraneoplastic Neurologic Disorders. 2024: 131–149. 10.1016/B978-0-12-823912-4.00019-0 38494274

[8] Varadan B, Shankar A, Rajakumar A, *et al*. Acute hemorrhagic leukoencephalitis in a COVID-19 patient—A case report with literature review. Neuroradiology. 2021;63(5):653–61. 33575849 10.1007/s00234-021-02667-1 PMC7878029

[9] Lim YY, Mormino EC. Alzheimer’s disease neuroimaging initiative. APOE genotype and early β-amyloid accumulation in older adults without dementia. Neurology. 2017;89(10):1028–34. 28794245 10.1212/WNL.0000000000004336 PMC5589795

[10] Liu C-C, Zhao N, Fu Y, *et al*. ApoE4 accelerates early seeding of amyloid pathology. Neuron. 2017;96(5):1024-1032.e1023. 29216449 10.1016/j.neuron.2017.11.013 PMC5948105

[11] Raulin A-C, Doss SV, Trottier ZA, *et al*. ApoE in Alzheimer’s disease: pathophysiology and therapeutic strategies. Mol Neurodegener. 2022. 10.1186/s13024-022-00574-4. 36348357 10.1186/s13024-022-00574-4 PMC9644639

[12] Smith EE, Charidimou A, Ayata C, *et al*. Cerebral amyloid angiopathy-related transient focal neurologic episodes. Neurology. 2021;97(5):231–8. 34016709 10.1212/WNL.0000000000012234 PMC8356377

[13] Charidimou A, Boulouis G, Frosch MP, *et al*. The Boston criteria version 2.0 for cerebral amyloid angiopathy: a multicentre, retrospective, MRI–neuropathology diagnostic accuracy study. Lancet Neurol. 2022;21(8):714–25. 35841910 10.1016/S1474-4422(22)00208-3 PMC9389452

[14] Smith EE, Cabot RC, Rosenberg ES, *et al*. Case 23–2024: a 78-year-old woman with rapidly progressive dementia. N Engl J Med. 2024;391(4):357–69. 39047245 10.1056/NEJMcpc2402488

[15] Theodorou A, Palaiodimou L, Malhotra K, *et al*. Clinical, neuroimaging, and genetic markers in cerebral amyloid angiopathy-related inflammation: a systematic review and meta-analysis. Stroke. 2023;54(1):178–88. 36453271 10.1161/STROKEAHA.122.040671

