# Supplementary figures and images for: Co-occurrence of lung adenocarcinoma with rapidly progressive dementia and multiple cerebral microbleeds: a case report
Source: J Med Case Rep. 2026 Feb 2;20:122. doi: 10.1186/s13256-026-05846-x (PMC12952105; doi:10.1186/s13256-026-05846-x)

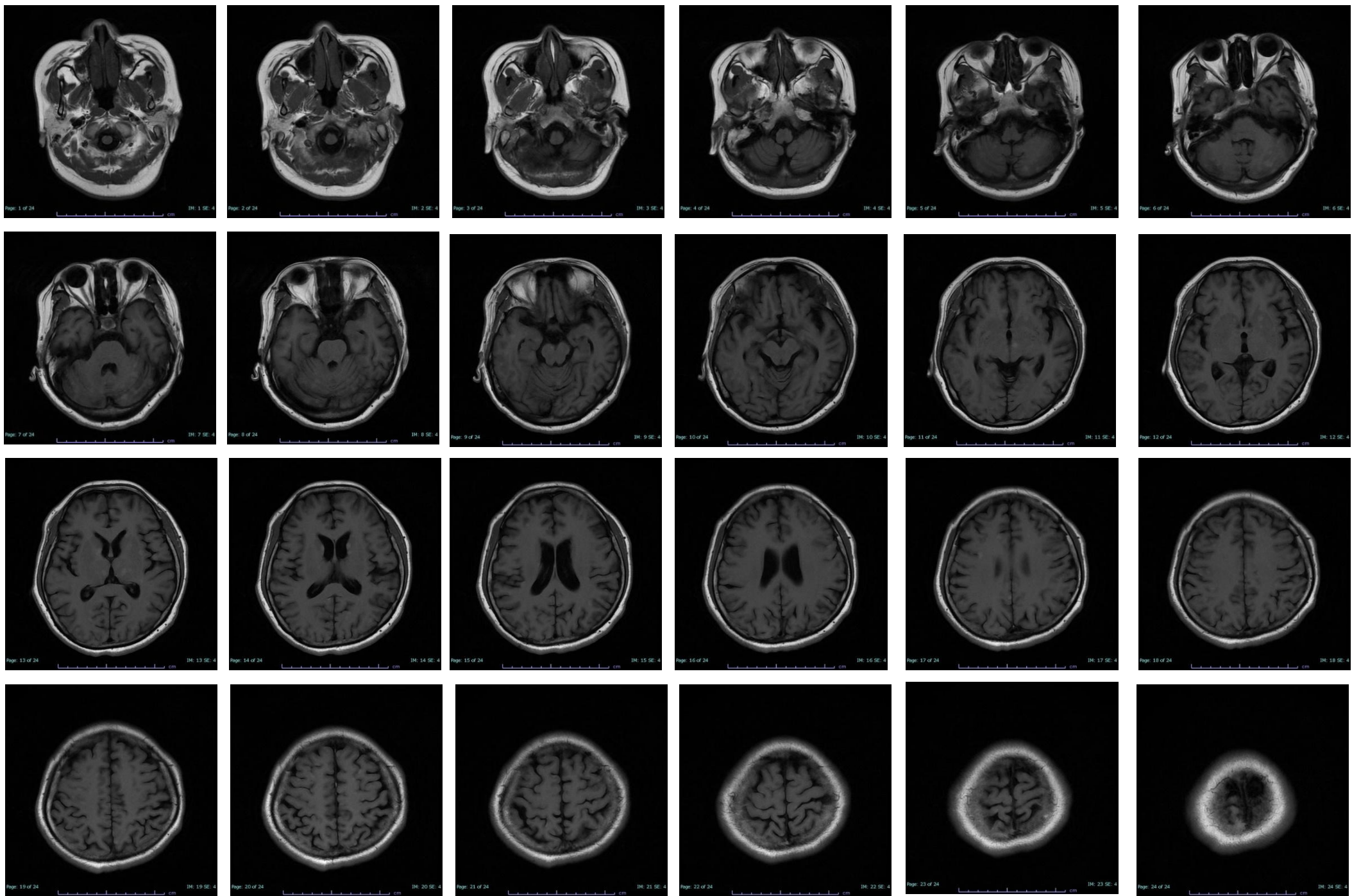

T1 flair

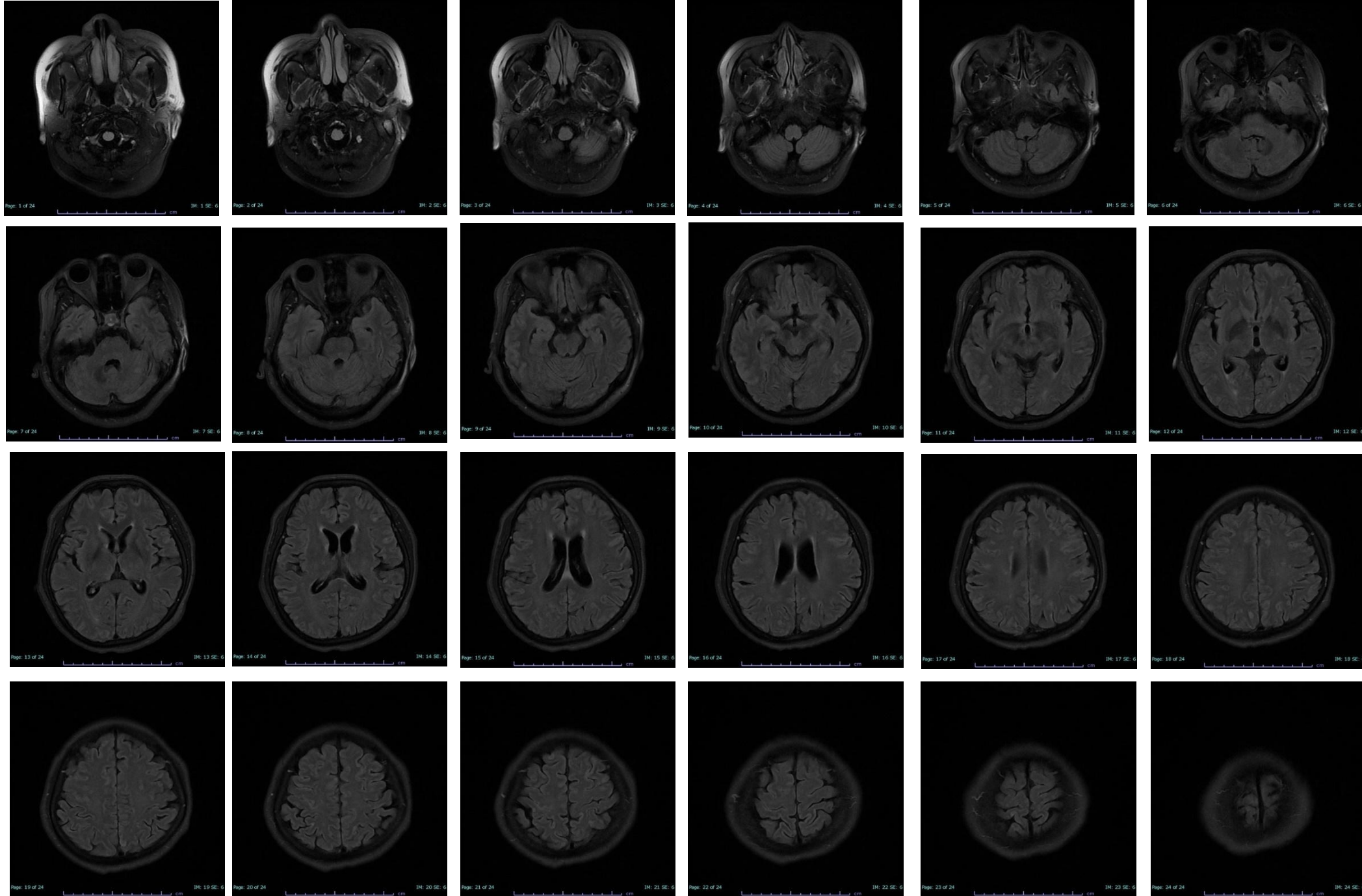

T2 flair

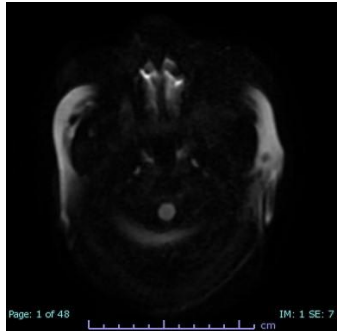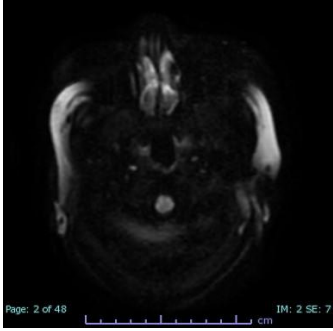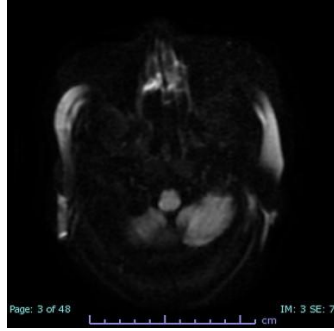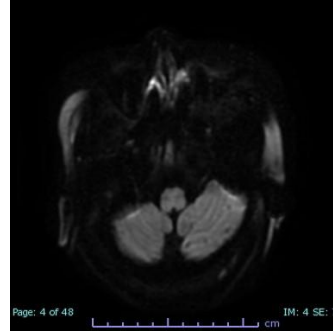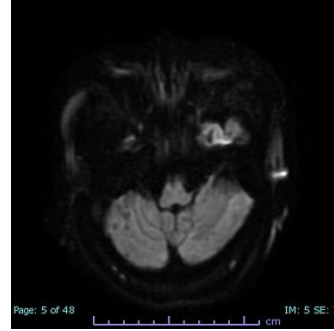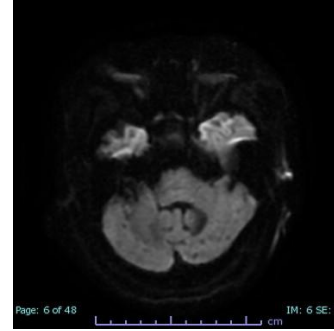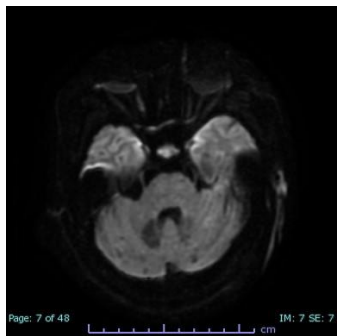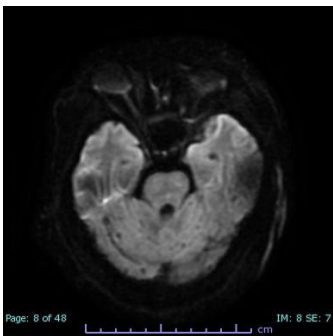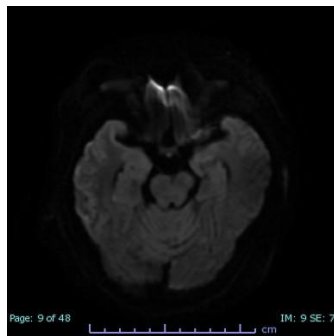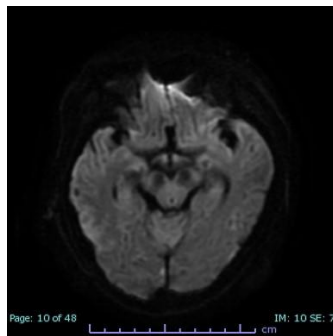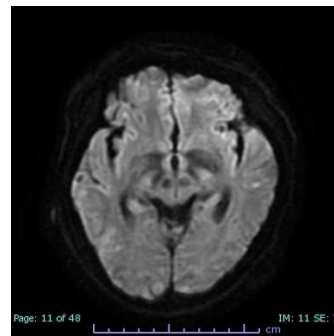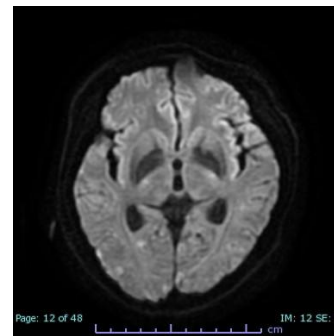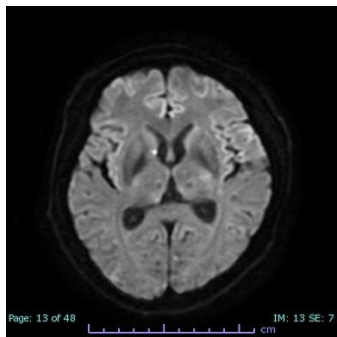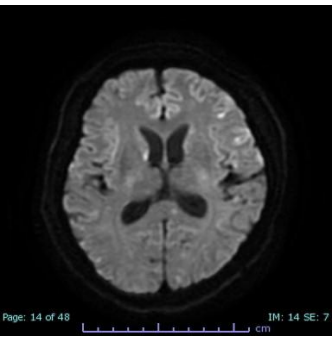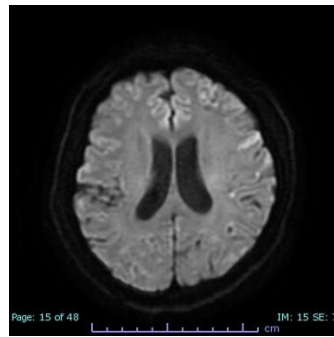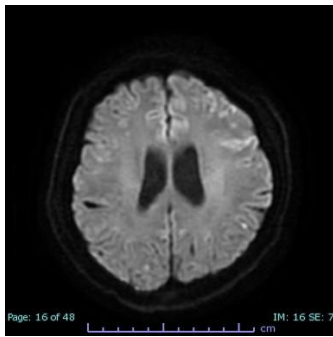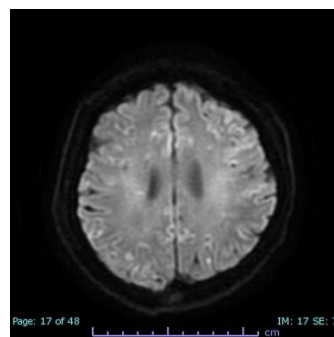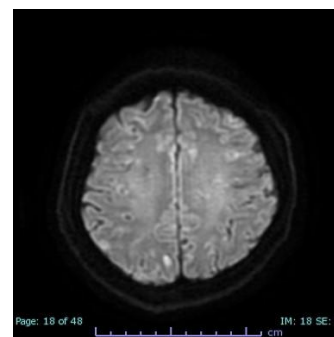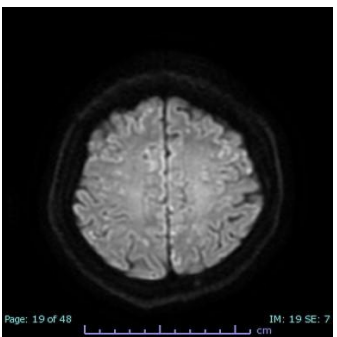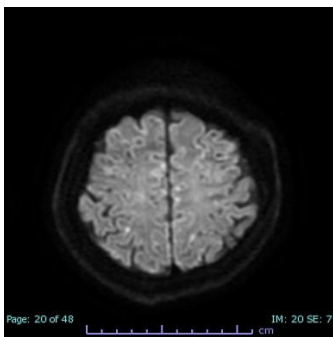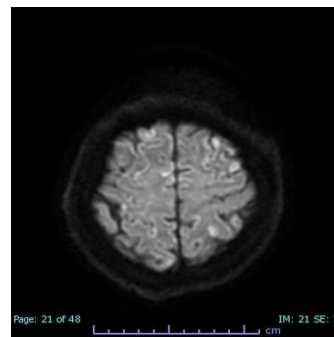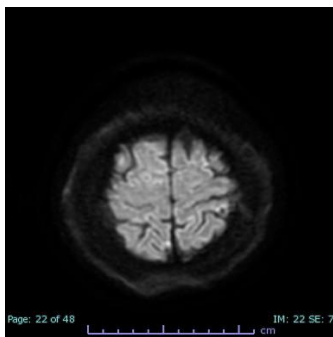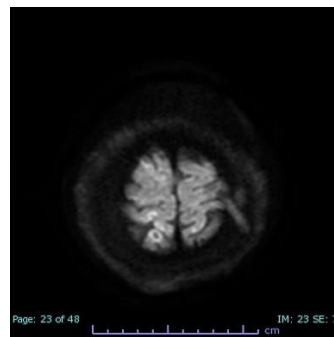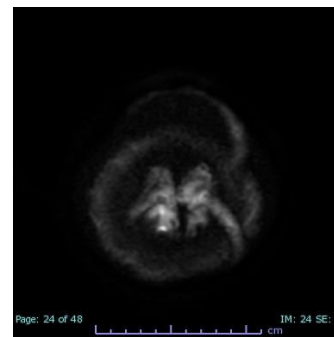

DWI

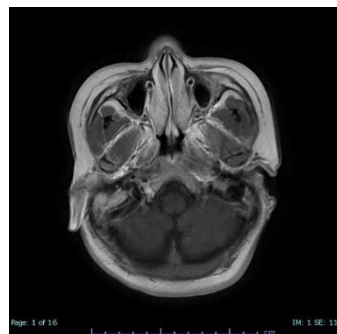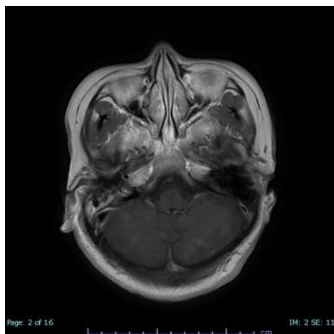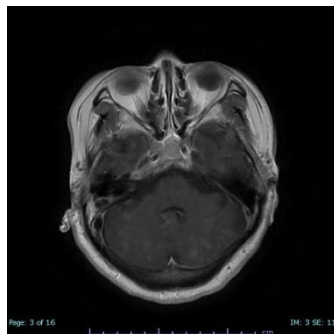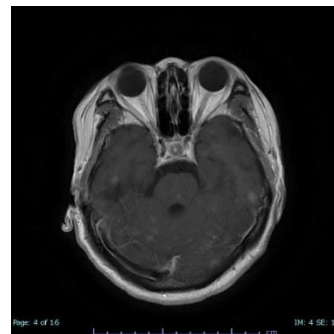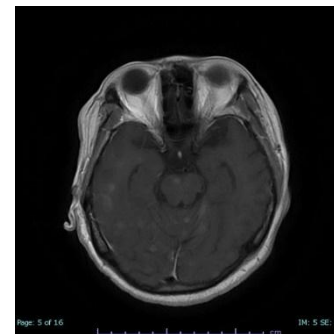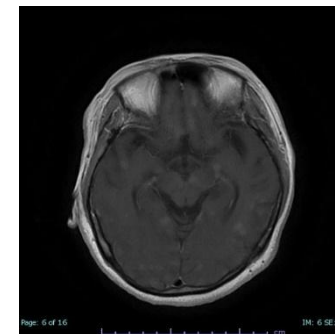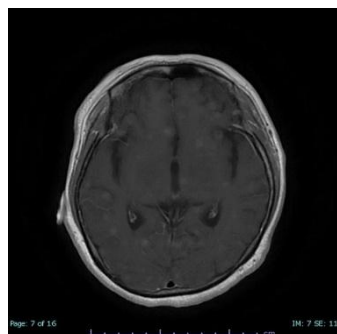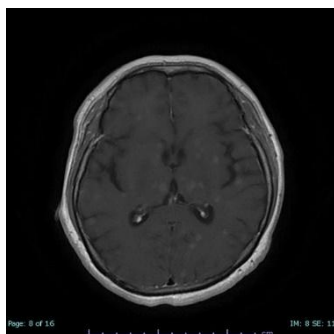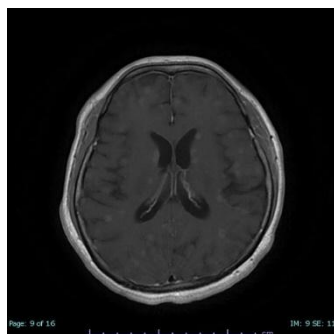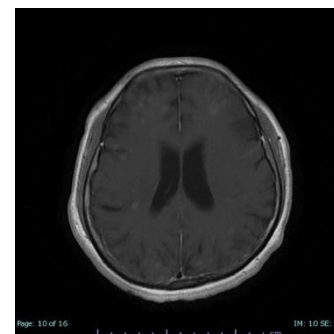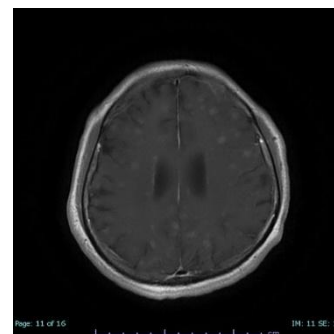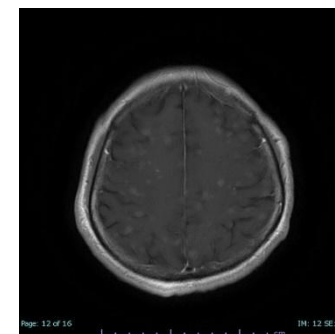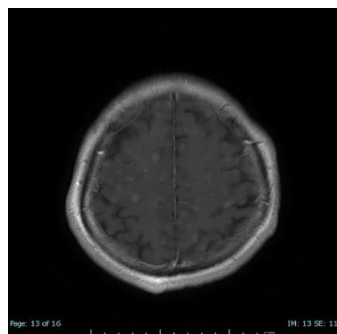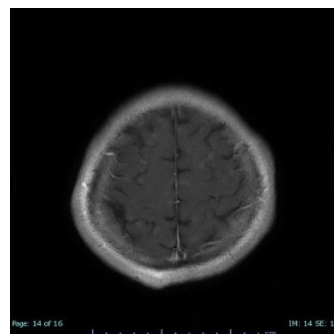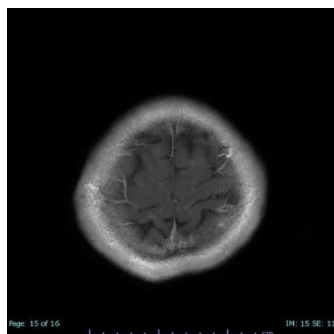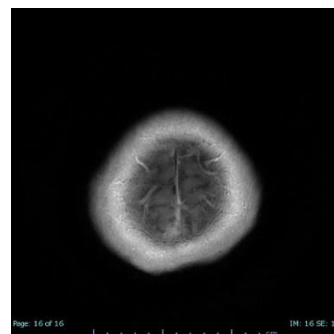

T1 enhances

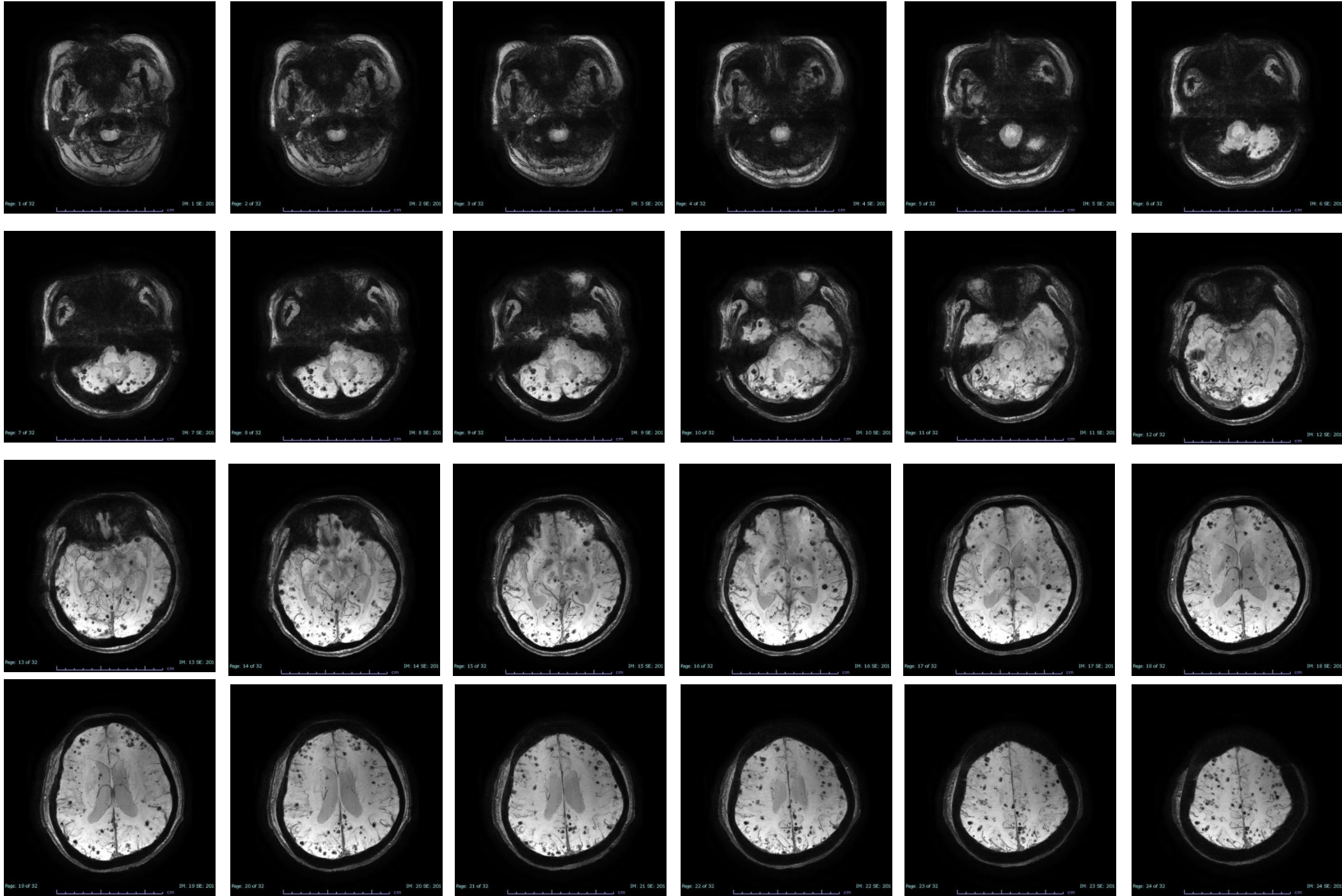

SWI

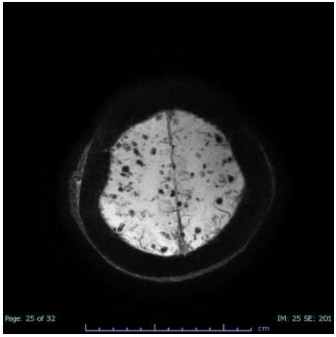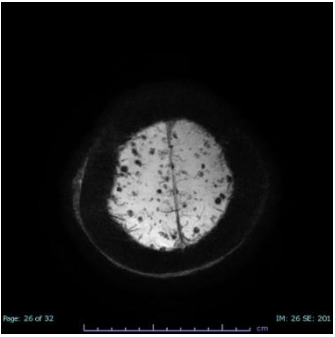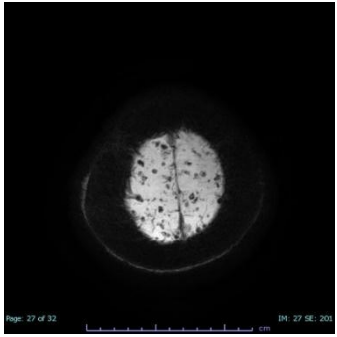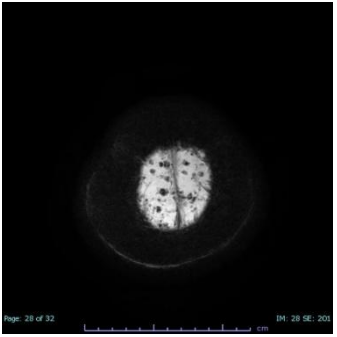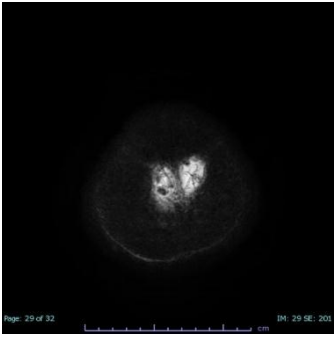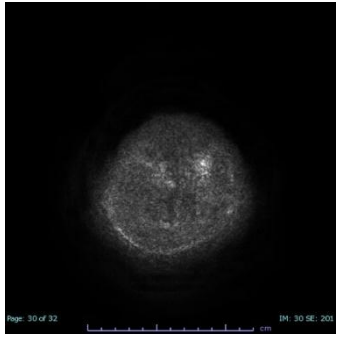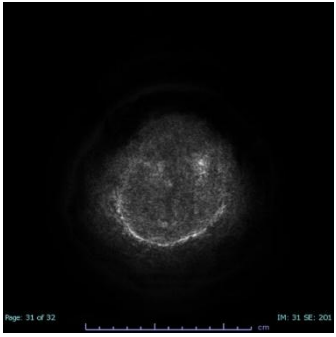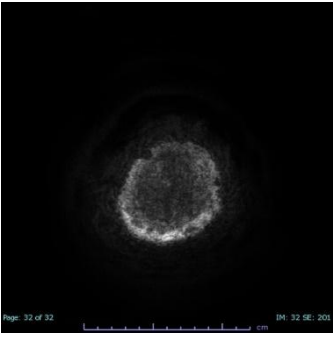

SWI

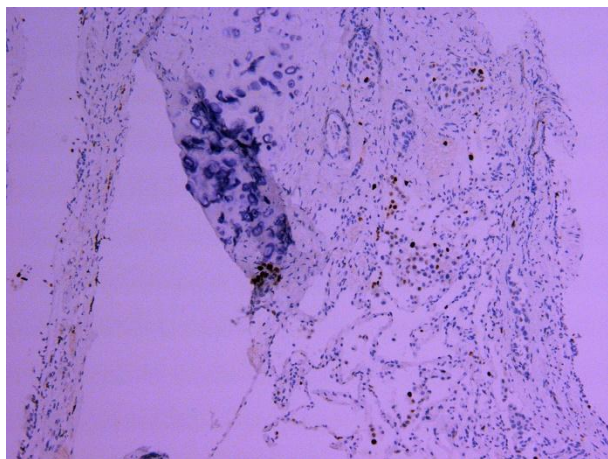

KI67

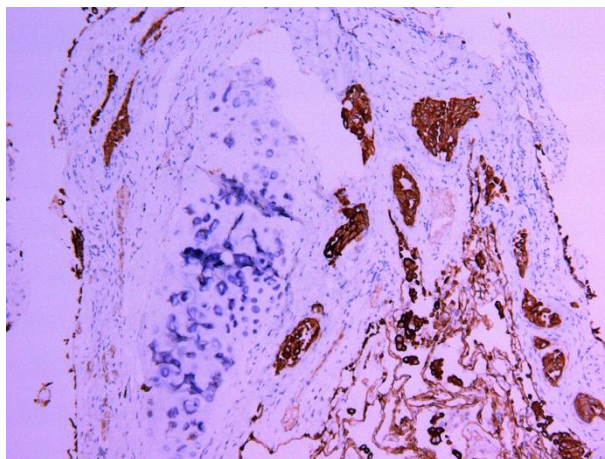

CK7

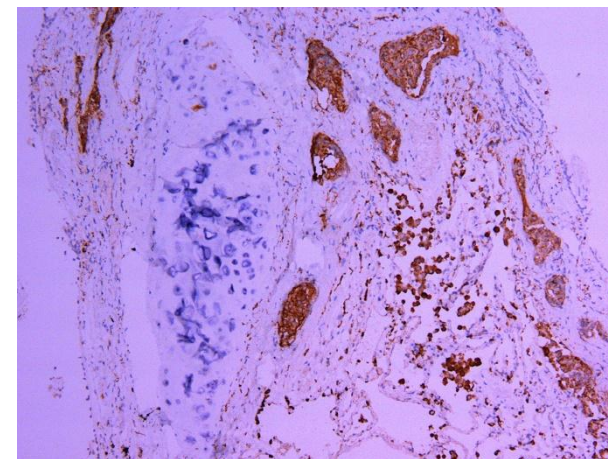

Napsin A

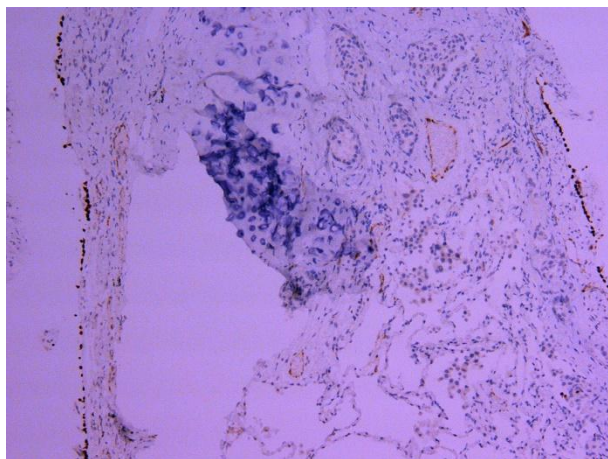

P40

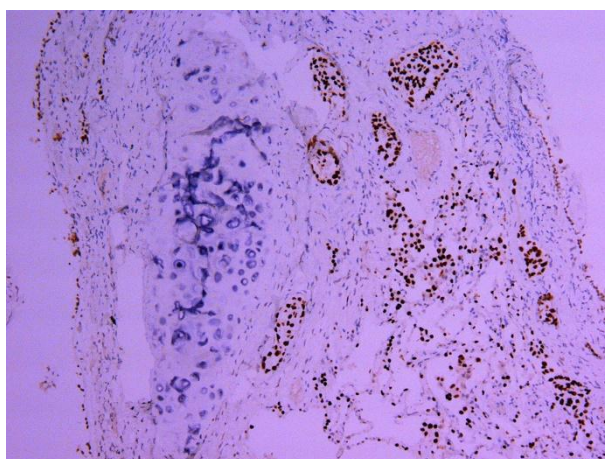

TTF1

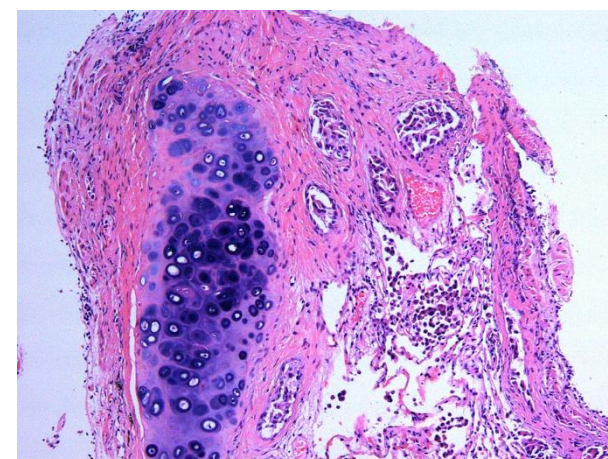

HE

pathological examination

Supplement: Supplementary file 1 — Supplementary material 1. [file 13256_2026_5846_MOESM1_ESM.pdf]
